# Effect of water stress and foliar application of chitosan and glycine betaine on lettuce

**DOI:** 10.1038/s41598-023-43992-0

**Published:** 2023-10-12

**Authors:** Ehab A. Ibrahim, Noura E. S. Ebrahim, Gehan Z. Mohamed

**Affiliations:** Vegetables Research Department, Horticulture Research Institute, Agricultural Research Center, 9 Cairo University St., Orman, Giza, Egypt

**Keywords:** Drought, Plant sciences, Plant stress responses

## Abstract

The present study investigated the effect of foliar application of chitosan at 150 ppm and glycine betaine at 700 ppm on lettuce plants cv. Balady grown under well-watered and water deficit conditions in terms of growth, yield, quality, and water usage efficiency. The study was conducted in Qalubia Governorate, Egypt, during the two seasons of 2020/2021 and 2021/2022 on clay soil. Results indicated that water-stressed plants had a reduction in plant fresh weight, plant height, leaf area, and total yield, chlorophyll content and relative water content, while they exhibited an increase in total soluble solids, nitrate, and proline contents as well as water-use efficiency in both seasons. The foliar application of chitosan or glycine betaine to lettuce significantly improved plant performance under limited and normal irrigation conditions in comparison with untreated plants. The maximum positive effect was for chitosan foliar application. Overall, the results of this study indicated that foliar application of chitosan or glycine betaine was a substitute technology for improving the lettuce yield and quality as well as increasing water use efficiency under both irrigation regimes, but may be more efficient in lettuce plants subjected to a water deficit.

## Introduction

Lettuce (*Lactuca sativa* L.) is one of the most widely cultivated and popular leafy vegetables worldwide. Its world production was 27.7 million tons^1^. Egypt produced 92,997 tons of lettuce from its estimated 3930 hectares of lettuce-growing land^2^. Lettuce is an important crop that plays a key role in people’s diets globally. It is a healthy source of minerals, fiber, vitamins, and bioactive molecules, including carotenoids, anthocyanins, and phenolic compounds^3^. Also, lettuce leaves are a rich source of antioxidants, fiber, and phytochemicals, which are important for reducing the risk of chronic diseases, such as diabetes, cancer, and cardiovascular disease^4^. Furthermore, lettuce plants naturally contain high levels of nitrate, which frequently exceed the permitted amount in the European Union^5^. The maximum acceptable nitrate concentration in winter-grown lettuce is 4000 mg kg^−1^ fresh weight^6^. So, reducing nitrate content can add value to lettuce plants.

Water deficit stress has emerged as one of the primary factors restricting vegetable output globally as a result of climate change, particularly in dry and semi-arid countries with limited water supplies. Indeed, the irrigation deficit preserved the growth productivity and quality of leafy vegetables. Water availability is essential for lettuce and has a significant impact on its growth, productivity, and quality^7,8^. Lettuce is extremely sensitive to drought and deficit irrigation due to its shallow root system and its leaves have relatively high water content (95%)^9^. It requires a lot of water (more than 95% of the soil's field capacity) to maintain its growth^10^. There is a mild drought if lettuce demand is between 75 and 90 percent of the field capacity^11^, and a severe drought if it is less than 50 percent^12^. However, lettuce may respond differently to low water supplies. Paim et al.^11^ and Thakur et al.^13^ found that the application of moderate drought stress, especially at the 80% level, represents an interesting strategy to improve the lettuce yield and quality. The appropriate drought stress reduced the nitrate content by 18% without affecting lettuce yield^14^. High lettuce productivity and minimal nitrate content were achieved with irrigation at 80% of crop evapotranspiration^15^. Plants exposed to excessive or deficient water accumulate more nitrate^16^.

Thus, finding practical technological solutions is crucial to reducing the negative effects of severe drought stress on plant production and quality in areas with limited water resources. Numerous strategies have been tested to improve plants' tolerance to drought. One of a solution is the use of compounds that can promote abiotic stress tolerance. Recently, there has been a lot of interest in applying natural compounds exogenously to improve a plant's tolerance to drought. Some of these compounds are amino acids such as glycine betaine^17^ and biostimulants such as chitosan^18,19^, which are still being explored.

Chitosan is a natural biopolymer, amino polysaccharide, and biodegradable material that is commercially produced from the exoskeleton of aquatic crustaceans^19^. Chitosan application at lower concentrations (0.2 g L^−1^) can mitigate the effect of drought stress and improve lettuce growth, but higher concentrations (0.4 g L^−1^ and 0.6 g L^−1^) inhibit the photosynthesis process and plant growth^20^. It is known to enhance plant growth regulators that protect plants from oxidative stress^21^.

Glycine betaine is an amino acid derivative that is naturally produced in many plant species. Abiotic stressors such as drought and salt stress enhance the synthesis of glycine betaine because it works as a compatible solute, maintaining the intracellular osmotic balance^17,22,23^ . However, many crop species, such as lettuce, cannot accumulate glycine betaine^24^. Exogenously applied glycine betaine in such crops promotes plant growth and improves plant water status, proline accumulation, water use efficiency, CO_2_ fixation, and stomatal conductance under normal and stressed conditions^23^. Exogenous glycine betaine treatment significantly reduced leaf nitrate content^25^. Moreover, glycine betaine has been shown to improve the plant’s tolerance to drought^26^. Thus, the exogenous application of glycine betaine is a promising strategy to improve the plant’s tolerance to drought^27,28^.

Despite the rising interest in using chitosan and glycine betaine in vegetable production systems, especially under abiotic stresses; there is a lack of research on how they affect the growth, production, and quality of lettuce plants at various irrigation levels. Thus, the goal of the present study was to examine how lettuce plants respond to chitosan and glycine betaine foliar spray under either normal irrigation or drought stress conditions in terms of growth, yield, quality, and water usage efficiency.

## Results

### Yield and its components

The irrigation water deficit significantly reduced plant fresh weight, plant height, leaf area, and total yield in comparison to well-watered plants in both seasons. In particular, the average yields of non-stressed plants were 109.96 and 107.76 ton ha^−1^ whereas plants subjected to drought had average values of 74.84 and 73.34 ton ha^−1^ in both seasons, respectively (Table 1). On the other hand, the foliar treatment of both chitosan and glycine betaine significantly enhanced these parameters compared to the control under limited and normal irrigation conditions. The highest significant values for plant fresh weight, plant height, leaf area, and total yield were obtained with chitosan treatment followed by glycine betaine, and the lowest values were noticed with control treatment in both seasons. Chitosan application was the best treatment under both deficit and normal irrigation (Table 1). Chitosan application increased yield by 4.13 to 4.21% compared to the control under normal irrigation, while under limited irrigation, yield increased by 26.71 to 27.15% during the first and second seasons, respectively.

**Table 1:** Effect of the interaction between irrigation regimes and foliar spraying of chitosan and glycine betaine (GB) on lettuce yield performance during the 2020/21 and 2021/22 seasons. Means followed with similar letters within the same column are not different significantly at *P* < 0.05 level of probability based on LSD test.

Treatments		Plant fresh wt (g)		Plant height (cm)		Leaf area (cm^2^ plant^−1^)		Total yield (ton ha^−1^)	
Irrigation	Foliar spraying	2020/21	2021/22	2020/21	2021/22	2020/21	2021/22	2020/21	2021/22
Well-watered	Water	647.3 c	634.3 c	37.8 ab	37.1 ab	5352.2 b	5245.2 b	110.0 b	107.8 b
	Chitosan	697.0 a	683.1 a	39.9 a	39.11 a	5541.3 a	5430.4 a	114.5 a	112.2 a
	GB	678.1 b	664.6 b	35.3 bc	34.6 bc	5494.2 a	5384.3 a	113.2 a	110.9 a
Drought	Water	473.7 f	464.2 f	31.6 c	30.9 c	3876.5 e	3798.9 e	74.8 e	73.1 e
	Chitosan	596.5 d	584.5 d	35.3 bc	34.6 bc	4794.6 c	4698.7 c	94.8 c	93.0 c
	GB	560.9 e	549.7 e	33.8 bc	33.2 bc	4469.8 d	4380.7 d	88.7 d	86.9 d
LSD at 0.05		6.68	6.55	4.35	4.26	135.93	133.2	1.41	1.33

### Physico-chemical properties

**Figure 1:**
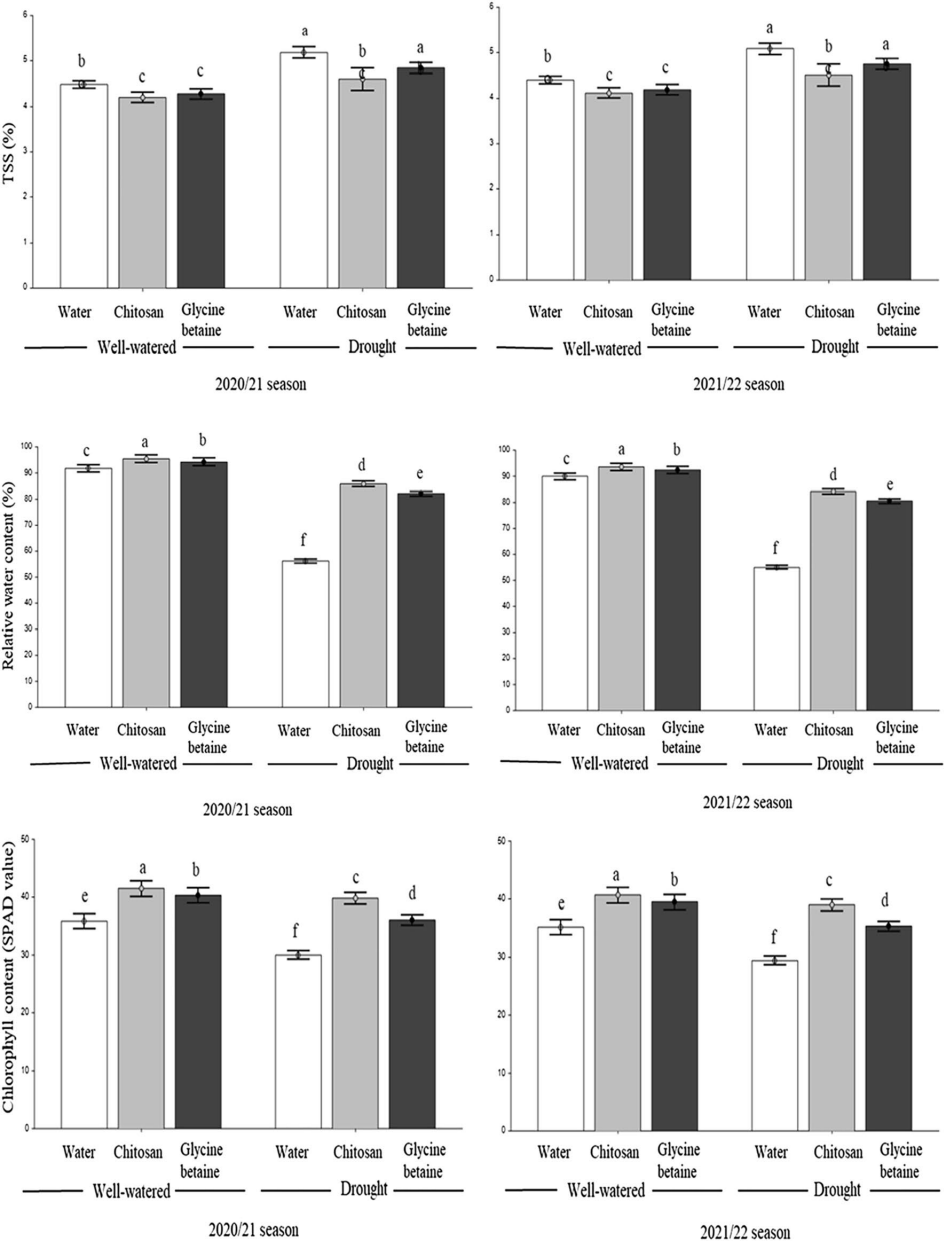
Effect of the interaction between irrigation regimes and foliar spraying of chitosan and glycine betaine on TSS, relative water, and chlorophyll content during the 2020/21 and 2021/22 seasons. Data are presented as the means ± SD (n = 3). Different letters mean significant differences among the treatments at *P* < 0.05 level.

**Figure 2:**
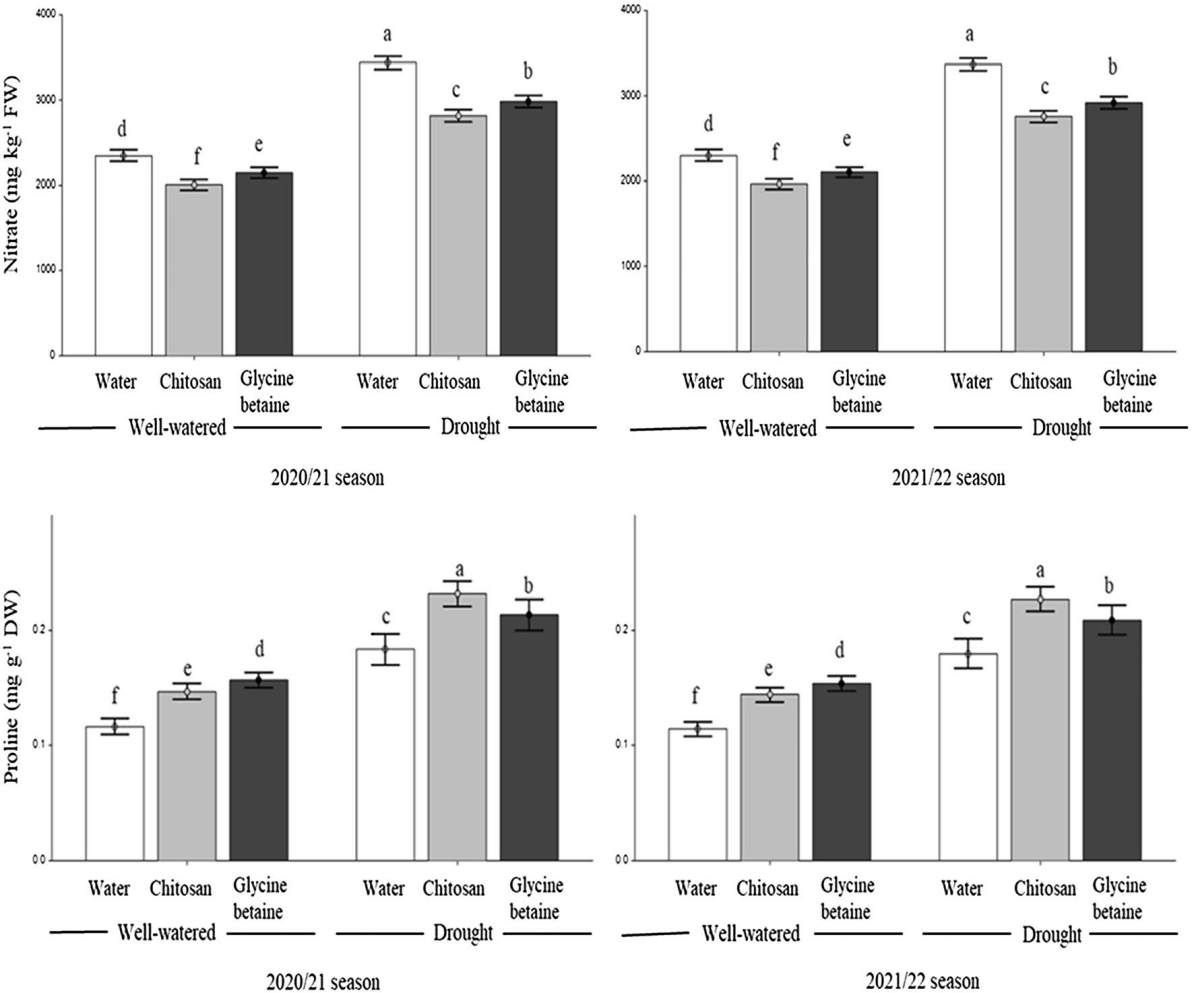
Effect of the interaction between irrigation regimes and foliar spraying of chitosan and glycine betaine on nitrate and proline content during the 2020/21 and 2021/22 seasons. Data are presented as the means ± SD (n = 3). Different letters mean significant differences among the treatments at *P* < 0.05 level.

The contents of total soluble solids (TSS), relative water, chlorophyll, nitrate, and proline as affected by irrigation regimes, and foliar application of natural compounds over two growing seasons are shown in Figs. 1 and 2.

Drought caused significant increases in the TSS, nitrate, and proline contents of lettuce leaves, while chlorophyll and relative water content were reduced in both seasons. In particular, the values of TSS were 4.53 and 4.44 in non-stressed plants and 5.25 and 5.15 in stressed plants in both seasons, respectively. Furthermore, the average values of nitrate concentration under stress-free conditions were 2380 and 2332 mg kg^−1^ FW, but they reached 3475 and 3406 mg kg^−1^ FW in stressed plants in both seasons, respectively. Similarly, proline levels in plants grown under constant water supply ranged between 0.120 and 0.118 mg g^−1^ DW, but they significantly increased in plants grown under water stress, reaching values of 0.190 and 0.186 mg g^−1^ DW in both seasons, respectively. On the other hand, foliar application of chitosan and glycine betaine enhanced the content of relative water, chlorophyll, and proline in comparison with control (spraying with tap water) under both irrigation regimes. The highest values were recorded by the chitosan application. Meanwhile, chitosan and glycine betaine significantly decreased the TSS and nitrate contents of lettuce leaves. Also, chitosan and glycine betaine applications enhanced TSS content in drought-stressed plants compared to unstressed plants. Similar results were observed in both seasons.

### Seasonal applied water

Irrigation levels significantly affected seasonal applied water in both growing seasons (Table 2). The highest values were recorded under normal irrigation treatment (regular watering), and the values were 4167.0 and 4083.7 m^3^ ha^−1^ in the first and second seasons, respectively. On the contrary, the lowest values were recorded under drought treatment, and the values were 2766.3 and 2710.9 m^3^ ha^−1^ in the first and second seasons, respectively. On the other hand, the foliar treatment of both chitosan and glycine betaine significantly reduced seasonal applied water (Table 2). The lowest values were obtained in plants grown under drought with the application of chitosan, and the values were 2439.1 and 2390.3 m^3^ ha^−1^ in the first and second seasons, respectively.

**Table 2:** Effect of the interaction between irrigation regimes and foliar spraying of chitosan and glycine betaine on seasonal applied water and water use efficiency during the 2020/21 and 2021/22 seasons. Means followed with similar letters within the same column are not different significantly at *P* < 0.05 level of probability based on LSD test.

Treatments		Total applied irrigation water (m^3^ ha^−1^)		Water use efficiency (kg m^3−1^)	
Irrigation	Foliar spraying	2020/21	2021/22	2020/21	2021/22
Well-watered	Water	4167.0 a	4083.7 a	26.40 f	25.87 f
	Chitosan	3819.6 c	3743.2 c	29.99 c	29.39 c
	Glycine betaine	4006.5 b	3926.4 b	28.26 d	27.69 d
Drought	Water	2766.3 d	2710.9 d	27.08 e	26.54 e
	Chitosan	2439.1 f	2390.3 f	38.93 a	38.15 a
	Glycine betaine	2663.3 e	2610.0 e	33.32 b	32.66 b
LSD at 0.05		10.03	34.2	0.48	0.47

### Water use efficiency

The water-use efficiency (WUE) values calculated for the various treatments in both seasons are summarized in Table 2. The well-watered treatment typically led to a decrease in WUE, whereas the stress treatments showed the highest WUE value. Chitosan followed by glycine betaine treatment displayed significantly higher WUE values compared to control treatment under well-watered and stress conditions. The highest WUE value was observed in plants grown under drought with the application of chitosan, and the values were 38.93 and 38.15 kg m^3−1^ in the first and second seasons, respectively.

## Discussion

Since lettuce's roots are small and its leaves contain a considerable amount of water, it is extremely sensitive to drought^10,16^. A water deficit significantly reduces lettuce weight and yield, and it also causes more oxidative damage^10,29,30^. In the present experiment, the reduction in water supply caused a reduction in plant fresh weight, plant height, leaf area, and total yield. These findings concur with those observed by Sayyari et al.^11^, Abd–Elrahman et al.^16^, Franzoni et al.^32^ and Hidalgo-Santiago et al.^33^ in lettuce plants produced under various water deficit conditions.

The detrimental effects of drought on lettuce plants may result from reduced water uptake by the leaves from the soil, increased dehydration, and decreased viscosity in the cells, all of which can have an adverse effect on vegetative growth traits, particularly plant height, leaf area, and fresh weight^10,16,31,32^. On the other hand, the plants could adapt to drought conditions by growing smaller, producing fewer leaves and stomata, and increasing the amounts of stress chemicals, including abscisic acid, which stimulates stomata closure and decreases the inflow of carbon dioxide and water transpiration while inhibiting photosynthesis^29,32–34^. Due to photosynthesis's restricted capability, all other biological processes are less effective, which causes less growth and production^7,35–37^.

The application of chitosan or glycine betaine is helpful in reducing biomass loss brought on by drought; therefore, it might be utilized in areas with a lack of water to help sustain lettuce production. These findings are consistent with those of other studies that found that foliar application of chitosan^20^ and glycine betaine^25^ improved lettuce vegetative growth. This improvement may be attributable to the fact that chitosan and glycine betaine were linked to enhancing stomatal conductance, photosynthetic rate, and antioxidant enzymes^19,38–40^.

The findings of this study revealed that drought stress increased the TSS, nitrate, and proline contents of lettuce leaves, but it also caused plants to have the lowest levels of chlorophyll and relative water content compared to non-stressed plants. In this respect, many studies have demonstrated how water stress affects plant chemicals^8,31,41,42^.

Changes in plant tolerance brought on by drought stress are linked to changes in metabolic traits. Plant tissue will accumulate free amino acids (especially proline) and soluble carbohydrates as a defense mechanism when there is a water shortage^42^. The accumulation of TSS could increase the plant's tolerance to drought stress^43^.

The significant proline accumulation in lettuce leaves may have an adapting role as an osmolyte generated under stressful conditions to reduce leaf osmotic potential and maintain cell turgidity. Also, proline has been proposed as a ROS scavenger that protects against oxidative damage to tissues^31^.

Foliar application of chitosan and glycine betaine, respectively, promoted TSS content and proline accumulation compared to control (spraying with tap water) under both irrigation regimes. It has been reported that chitosan and glycine betaine applications enhanced TSS content in drought-stressed okra plants^28^. Moreover, the high level of proline in stressed plants induced by chitosan and glycine betaine application might be an indication of enhanced plant tolerance to water stress^44,45^. The increased proline level caused by using chitosan may be attributed to a decrease in proline oxidation to glutamate and an increase in proline biosynthesis^44,46^.

Plants that were subjected to a water deficit had higher levels of nitrate in their leaves. These findings agree with those observed by Franzoni et al.^32^ and Abd-Elrahman et al.^16^, in lettuce plants grown under water deficit regimes. The high quantity of nitrate detected under water stress may be the result of diminished nitrate reductase enzyme activity^47^. Also, the function of nitrate as an osmotic regulator may be another factor contributing to this increase^25,48^. It has been reported that glycine betaine application decreased nitrate content^25^.

Plants that were subjected to drought stress had the lowest amount of chlorophyll compared to control plants^7^, resulting in reduced photosynthesis efficiency and plant growth^29,32,46^. However, chitosan and glycine betaine applications prevent these negative effects by increasing chlorophyll levels in water-stressed plants and increasing photosynthesis^28,39^.

The increase in irrigation water applied under normal irrigation may be due to the increase in direct evaporation. As a result, more seasonal irrigation water is used under regular irrigation than it is under drought treatment during the two growing seasons of lettuce. These findings are in line with those reported by Mostafa et al.^49^. Moreover, the foliar application of chitosan and glycine betaine considerably decreased the seasonal applied water. They might function as antitranspirants, allowing the plant to absorb more water over an extended period of time^50^. Similar results were reported by studies that show that chitosan and glycine betaine application under water-stressed conditions can increase stomatal conductance and raise stomatal resistance due to stomata closure, which in turn improves the plant's water status^23,26,38^.

The findings of the current study demonstrated that the WUE of lettuce increased as the severity of the water stress increased. In this respect, Mostafa et al.^49^, Khani et al.^31^ and Sesveren and Taş^51^ revealed that lettuce plants had greater irrigation water use efficiency when less irrigation water was administered. The WUE can be increased by increasing the yield or by decreasing the frequency of irrigation and total irrigation water input. In water-limited cases, it is widely accepted that WUE is positively correlated with yield and drought tolerance^39^. In the present study, using chitosan and glycine betaine on plants under drought significantly increased WUE values compared to the control. The application of these compounds probably improves the water status of lettuce plants and allows a greater photosynthesis rate under water-stressed conditions. This finding agreed with those obtained by Lin et al.^40^.

## Conclusions

The findings of this study show that lettuce growth, yield, and physiological traits are adversely affected by drought stress. The foliar application of chitosan at 150 ppm and glycine betaine at 700 ppm three times at 20, 35 and 45 days after transplanting increased the lettuce yield and quality, as well as improved water use efficiency under regular watering and drought stress. Thus, the practical applications of these compounds could be a promising technology used to reduce the harmful effect of drought stress on lettuce and improve water relations. Further studies with other crops by testing different application modes should be performed in the future.

## Materials and methods

### Site description

A field study was conducted at the Qaha Vegetable Research Farm (30°17′ 25" N, 31°11′ 49" E) in Qalubia Governorate, Egypt, during the 2020/2021 and 2021/2022 growing seasons.

**Figure 3:**
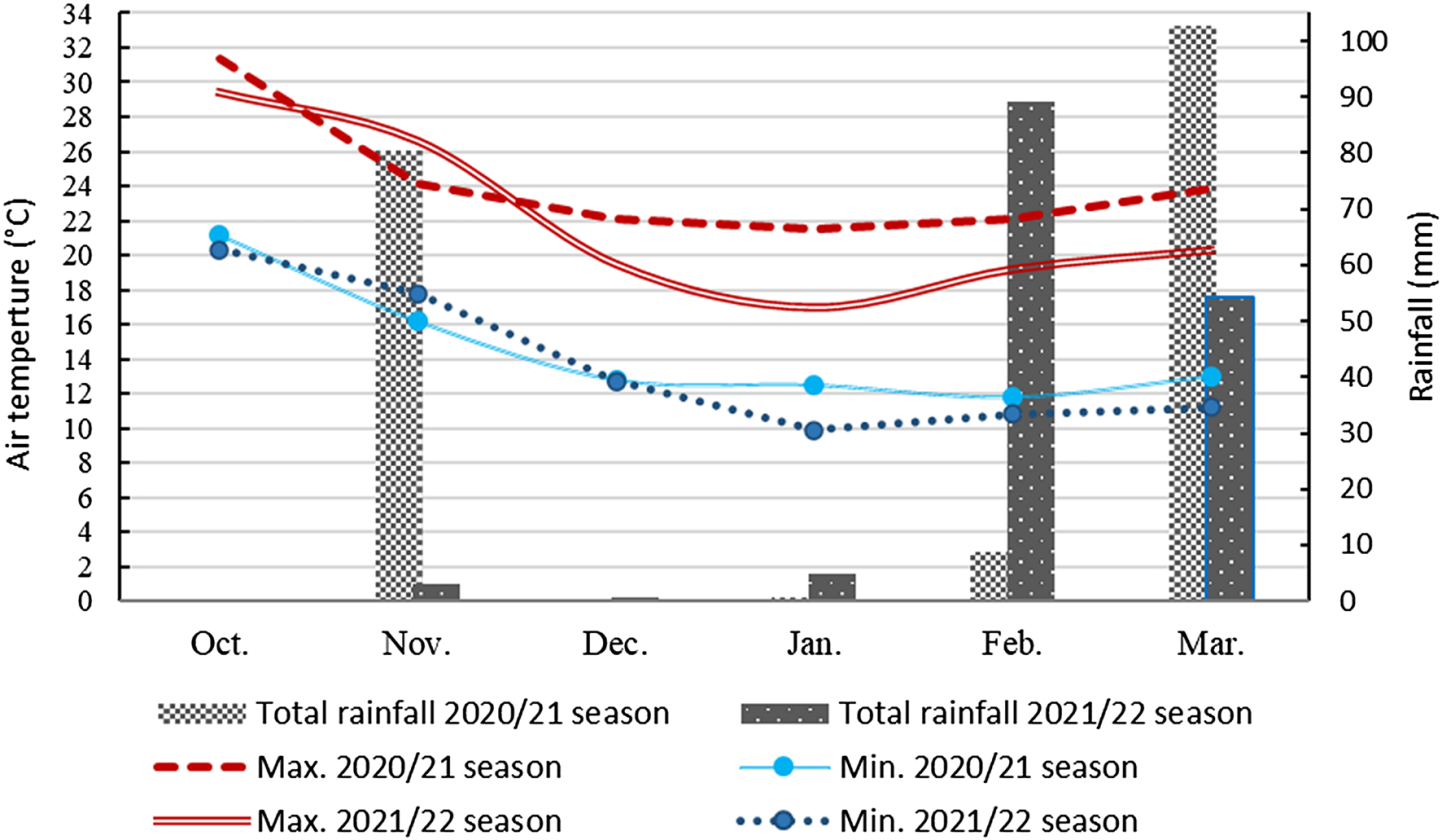
Monthly temperature and rainfall during the 2020/21 and 2021/22 seasons.

The physical and chemical parameters of the experimental soil at a depth of 0–30 cm were determined using the methods outlined by Page et al.^52^ and Klute^53^, and the findings are shown in Table 3. The weather conditions for the two lettuce growing seasons are shown in Fig. 3.

**Table 3:** Physical and chemical analysis of the experimental soil before planting during the two winter seasons of 2020/2021 and 2021/2022.

Properties	Values		Properties	Values	
	2020/2021	2021/2022		2020/2021	2021/2022
Sand (%)	23.14	21.16	Available N (ppm)	66.74	71.81
Silt (%)	28.59	29.11	Available P (ppm)	5.23	5.86
Clay (%)	48.27	49.73	Exchangeable K (ppm)	59.60	58.42
Texture class	Clay	Clay	Field capacity (%)	46.25	45.73
CaCO3	2.57	2.44	Wilting point (%)	24.21	23.84
OM (%)	1.36	1.42	Available water (%)	22.04	21.89
pH*	7.8	7.7	Bulk density (g/cm^3^)	1.22	1.21
EC (dSm^−1^) (1:10)	2.2	2.1

### Experimental design and crop management

Lettuce seedlings "cv. Balady" were transplanted when they were five weeks old, on the 30th and 25th of October in both seasons, respectively. A split-plot in randomized complete blocks design with three replicates was followed. In each experimental sub-plot, five rows were used. Each row measured 4 m long and 60 cm wide. The transplants were placed on both sides of the ridges, with 20 cm between them. Two guard ridges separated each treatment. The main plots were assigned to irrigated (well-watered (irrigation every 10–12 days) according to the recommendation of Egyptian Ministry of Agriculture) and drought stress conditions (half of the irrigations compared to the irrigated ones, i.e., missing alternate irrigation). Subplots were devoted to the three foliar treatments (tap water as a control, chitosan at 150 ppm, and glycine betaine at 700 ppm).

Irrigation treatments were applied after the initial irrigation that was done ten days following the transplant. The total number of irrigation events was 8 and 5 for well-watered and drought treatments, respectively. Foliar treatments were applied three times at 20, 35, and 45 days after transplanting. The plants were sprayed with a hand sprayer until drop-off in the afternoon.

Chito-Care®, a local commercial chitosan (2-amino-2-deoxy—d-glucosamine) product with an 85% degree of deacetylation, was used. Chitosan and glycine betaine were dissolved in tap water to achieve the targeted concentrations. Nitrogen in the form of ammonium sulfate (20.6% N) was applied at a rate of 143 kg N ha^−1^, phosphorus in the form of calcium superphosphate (15.5% P_2_O_5_) was applied at a rate of 92 kg P_2_O_5_ ha^−1^ and potassium in the form of potassium sulfate (48% K_2_O) was applied at a rate of 114 kg K_2_O ha^−1^. These fertilizers were applied in two equal doses to all plots in the third and fifth weeks after seedling transplanting. Other recommended agricultural practices for cultivating lettuce plants were followed.

### Data and measurements

The lettuce plants were harvested 85 days after transplanting. Nine plants were randomly selected from the three central rows of each subplot for measuring plant fresh weight, plant height, and leaf area per plant with a portable leaf area meter (Li-3100, USA). The TSS% values were measured using a Carl-Zeiss hand refractometer.

To quantify total chlorophyll, the middle region of a young, fully grown leaf on six leaves per plot was examined using a handheld chlorophyll meter device (SPAD-502, Minolta, Sakai, Osaka, Japan). SPAD data can be used to forecast leaf carotenoids and nitrogen levels^54^. The percentage of relative water content (RWC) in leaves was calculated according to Barrs and Weatherley^55^. The 20 leaf disc samples (10 mm in diameter) were weighed and soaked in distilled water for 24 h. Finally, samples were removed from the water, dried, and weighed right away to get a fully turgid weight. The samples were then dried in an oven at 65 °C for 48 h, and their dry weights were recorded afterward. The RWC was determined as follows:

1 $${\text{RWC}}\;\left( \% \right) = \left[ {\left( {{\text{W}} - {\text{DW}}} \right)/\left( {{\text{TW}} - {\text{DW}}} \right)} \right] \times {1}00$$

Where W is a sample of fresh weight, TW is a sample of turgid weight, DW is a sample of dry weight.

Nitrate was extracted using 2% acetic acid and determined according to Singh^56^. A 100-g sample of leaves was weighted and then oven-dried at 70 °C until the weight was consistent. Thereafter, their dry weights were recorded to calculate their dry matter percentages. Proline content was measured as mg g^−1^ DW at 520 nm using a spectrophotometer, according to Bates et al.^57^. Plants in plots were weighted in kg per plot to determine yield, which was then converted to an Mg ha^−1^ estimate.

According to Israelson and Hansen^58,59^, water consumption use was calculated using the difference in soil moisture content before and after irrigation:

2 $$Cu \, = \, D \times Bd \times 10000 \times \left( {\theta_{2} - \, \theta_{1} } \right)/100$$

where Cu is the water consumptive use m^3^//ha, D is the depth of the soil (30 cm), Bd is the soil bulk density (g cm^−3^), θ_1_ represents the soil moisture prior to irrigation (% by weight), and θ_2_ represents the soil moisture after 48 h from irrigation (% by weight).

The seasonal applied water is the sum of the figures obtained for each irrigation application. Water use efficiency was estimated for each treatment by dividing the yield (kg ha^−1^) by the total irrigation water supplied (m^3^ ha^−1^)^59^.

### Data analysis

Data were analyzed using the analysis of variance technique, and differences between individual pairs of treatment means were tested using the Least Significant Difference (LSD) at *p* < 0.05 by Costat 6.29 computer program (CoHort Software), according to Snedecor and Cochran^60^. Microsoft Excel® 2013 and the statistical program Number Cruncher Statistical System were used to tabulate and analyze the data.

## Research involving plants statement

This study was developed with commercial seeds, therefore nonexotic or at risk of extinction, under controlled conditions, meeting all institutional, national and international guidelines and legislation for cultivated plants.

## Data Availability

All data generated and/or analyzed during this study are available from the corresponding author on reasonable request.
